# Minimum dietary diversity and associated factors among children aged 6-23 months in Enebsie Sar Midir Woreda, East Gojjam, North West Ethiopia

**DOI:** 10.1186/s40795-022-00644-2

**Published:** 2022-12-20

**Authors:** Dejenu Assefa, Tefera Belachew

**Affiliations:** 1 Institute of Technology, Food Science Department, Bahir Dar University, Bahir Dar, Ethiopia; 2 Department of nutrition and dietetics, Faculty of Public Health, Jimma University, Jimma, Ethiopia

**Keywords:** Minimum dietary diversity, Infant and young child feeding, Associated factors, Enebsie Sar Midir

## Abstract

**Background:**

Dietary diversity has been recognized as a significant component of high-quality diets for children’s growth and development. Poor infant and young child feeding practices in the first one thousand days of age is the major contributor of malnutrition that leads to failure to thrive to their age, in low-income countries including Ethiopia. It causes long-term consequences of chronic malnutrition, including as stunting, has an impact on intellectual development, and raises the risk of several infectious diseases and death. There was no research done on the dietary diversity of the children in the study area.

**Objective:**

The main objective of this study was to assess minimum dietary diversity and associated factors among children aged from 6-23 months in Enebsie Sar Midir Woreda.

**Methods:**

A community-based cross-sectional study design was used to assess the minimum dietary diversity and associated factors among children aged 6–23 months in Enebsie Sar Midir Woreda, East Gojjam, North West Ethiopia. A total of 512 Mothers/caregivers of children of 6–23 months old in the community were included in the study using a systemic sampling technique. Data were collected by the interviewer-administered structured, pre-tested questionnaire. A 24-hour recall method was used to capture the dietary intake of children during the previous 24 h period before the interview. The data were coded and entered into SPSS for windows version 20 and analyzed after cleaning. Descriptive statistics and bivariate and multivariable logistic regression models were used to isolate independent predictors of minimum dietary diversity. All tests were two-sided and *P* < 0.05 was used for declaring statistical significance.

**Results:**

The overall prevalence of minimum dietary diversity was 18.2% (CI: 14.8, 21.7). The dominant food groups consumed were roots, grains, and tubers. While consumption of vitamin A-rich fruits and vegetables and other vegetables were relatively low.

On multivariable logistic regression model, after adjusting for other variables, availability of cow’s milk at household (AOR = 17.27; 95% CI: 6.73, 44.44), cultivating vegetables (AOR = 3.2; 95% CI: 1.05, 9.8), availability of farmland (AOR= 10.15, 95%CI: 1.78, 57.93) and number of animals (AOR = 6.46; 95% CI: 1.97, 21.12) were significantly associated with minimum dietary diversity.

**Conclusion:**

The proportion of infant and young children aged between 6-23months receiving minimum dietary diversity score is low compared with a study conducted in Addis Ababa. Availability of both animal and plant-source foods from household production was positively associated with practicing the minimum dietary diversity among children implying the need for strengthening nutrition-sensitive agricultural practices.

## Background

Adequate nutrition allows children to achieve the proper milestones, to develop, grow, attain learning, and play, while undernutrition negatively affects children’s total growth and development. Stunting is one of the main reasons that affect the children mentally and physically leading to impairment of their physical growth and development of their full intellectual potential [1].

The Ethiopian government has been implementing the national nutrition program, the Infant and Young Child Feeding (IYCF) strategy, and has developed the Seqota Declaration, a multi-sectoral nutrition intervention plan to address the underlying, basic, and immediate causes of malnutrition in Ethiopia by 2030 [2]. Despite the fact that the Ethiopian government has undertaken a number of interventions, including the transmission of knowledge about the value of diverse food through various media, the practice of providing diversified food remains low, as evidenced by many studies [3–11].

“Minimum Dietary Diversity: is the consumption of four or more food groups from the seven food groups for higher dietary quality and to meet daily energy and nutrient requirements of the seven recommended food groups namely: grains, roots and tubers; legumes and nuts; dairy products; flesh foods (meat, fish, poultry and organ meats); eggs; vitamin-A rich fruits and vegetables; other fruits and vegetables” [12].

According to a cross-sectional study conducted in Mekele, inappropriate complementary feeding practices remain a major public health problem in many poor nations, with many children becoming victims of malpractice [13]. Only about a third of children aged 6 to 23 months met the bare minimum of dietary diversification [14]. A health facility-based cross-sectional study conducted in Addis Ababa on minimum dietary diversity shows that children with minimum dietary diversity were 59.9%. Mother’s educational attainment and a higher household monthly income were positively associated with the minimum dietary diversity practice. Similarly, mothers’ knowledge on dietary diversity and child feeding were positively associated with minimum dietary diversity o0f children [11]. A study conducted on the nutritional status and dietary diversity of children below two years in Kasungu and Mzimba, Malawi have shown Minimum dietary diversity were 60% [15].

A community-based cross sectional cluster survey carried out in northern Ghana shows 34.8% received minimum dietary diversity (≥ 4 food groups) [16]. Several cross sectional survey conducted in Asia and China have shown approximately half of all children met the MDD, varying from 47·7% in Cambodia to 58·2% in Indonesia, 24·6% in Myanmar and breastfeeding (51.7%) than non-breastfeeding (71.9%) in China [17, 18].

In Ethiopia, over the past 15 years, remarkable decline in child under-nutrition had been observed. Childhood malnutrition still remains a major public health challenge in Ethiopia. As of 2019 Ethiopian Mini Demographic and Health Survey reported that 37%, 21% and 7% of under five years children were stunted, underweight, and wasted, respectively [19]. However, this is hypothesized to be even worse in Amhara Region (the study area) due different social factors such as fasting animal source foods, which also obliges the child to fast.

A meta-analysis conducted in Ethiopia on dietary diversity and feeding practice among children age from 6 to 23 months result showed that the pooled prevalence of dietary feeding practice among children age 6–23 months in Ethiopia was 23.25% . In the subgroup analysis, the lowest prevalence was observed in Amhara region (study area) (12.58%) [20].

Best search of literature showed that Minimum Dietary Diversity and associated factors have not been widely studied so far among children 6–23 months in Enebsie Sar Midir Woreda. Thus, this study identified the minimum dietary diversity and associated factors in Enebsie Sar Midir Woreda.

## Methods

### Study setting and subjects

The study was conducted in Enebsie Sar Midir Woreda, East Gojjam Zone, North West Amhara. Enebsie Sar Midir is one of the woredas in East Gojjam Zone ANRS. The Woreda is located 365 kilo meters far from Addis Ababa, the capital city of Ethiopia and 182 km far from the capital city of Amhara National Regional State, Bahir Dar. The organizational structure of the Woreda includes four urban and 31 rural kebeles. There are eight health centers /HCs/ and one governmental Hospital in the woreda.

Based on the 2007 national census conducted by the Central Statistical Agency of Ethiopia (CSA), the woreda has a total population of 133,855, of whom 66,139 were men and 67,716 women; 12,259 or 9.16% were urban inhabitants.

#### Study design and study period

Cross sectional community-based study design was conducted from January to February 2020to assess minimum dietary diversity.

#### Source population

 All children aged 6–23 months along with mothers or care givers who located in Enebsie Sar Midir Woreda were the source population.

#### Study population

All children aged 6 months to 23 months along with mothers or care givers drawn from the selected kebeles were the study population.

#### Sample size determination

A single population proportion estimation formula was used to calculate the sample size. With a precision (d) of 3%, a confidence level of 95% (z = 1.96), and an expected prevalence of adequate dietary diversity of 13% [21], a sample size of 482 was calculated; however, when a 10% non-response rate was added, the total sample size was 530.

### Sampling method

Systematic sampling was used to select the study subjects in the community. As households were sampling units the sampling interval was obtained by dividing the total number of households of 19,122 by the sample size. There are 34 kebeles in the woreda, 30 of which are located in the rural woreda and 4 in the urban woreda. Thirteen kebeles, or around 30% of all kebeles, were included in the study, with 11 from rural areas and two from urban areas.

The first household was randomly selected within the interval of ten using lottery method. Then a bottle was tossed in the middle of each kebele after deciding to follow the head of the bottle. Then the households were counted in the direction of the head of the bottle to identify the first household. After that the next households were selected by adding the interval. In the even bet where there is no eligible person within the selected household, the immediate next household was visited and this continued until the whole sample size was fulfilled.

#### Inclusion criteria

All mothers and care givers with infants six months to 23 months of age were included in the study.

#### Exclusion criteria

Seriously ill mothers, non-consenting mothers were excluded from study.

### Study variables

To analyze the data, the study focused on the following dependent and independent variables. The dependent variables of this study were Minimum Dietary Diversity, and Independent Variables like Socio Demographic Characteristics, Economic characteristics of parents, Agricultural factors and Wealth Index.

### Data collection procedure

#### Data collection tool

The data collection tool was structured interview questionnaire which had factors for minimum dietary diversity.

#### Data collection procedure

The data collection procedure started from identifying the study subjects and selected randomly who was included in the study. The data collection was conducted by using 24-hour recall method in the community from mothers or care givers. All participants who fulfill the criteria were participated. Then oral informed consent was conducted.

#### Data collector

The data collectors were grade 10 and 12 complete persons who have previous data collection experience in health care program areas.

Before the data collection, the principal investigators had given basic training about the questionnaire. Thus, all data collectors know about the objectives of the study, the data collection approach, the discipline need to have during data collection and other important issues related to the problem.

#### Supervisors

The principal investigator, and health extension workers were recruited the supervisor purposively. These persons were familiar about the overall information needed, about the purpose of this research as a supervisor to guide the data collectors, about the time of collection, how to manage the study subjects during data collection procedure, and fix the time from when to when completed questionnaire received. The principal investigator closely followed the data collection procedure. Completed questionnaires were rechecked for completeness and consistency starting from the beginning of the data collection to provide feedback for the next steps of the data collection throughout the of the data collection time.

### Data quality control

The structured interview questionnaire was adapted from WHO IYCF indicators and other related scholars to the specific settings considering the objective of the study [22, 23]. The questionnaire was modified according to the study variable included and specific context. The questionnaire was prepared in English first and then it is translated in to Amharic by experts who work on this area before data collection. Pretest was done on 5% of the total sample of the study subject to ensure that the language and contents are culturally suitable, acceptable, and clearly understandable for the participants. Before data collection took place necessary corrections were made on the questionnaire to ensure the quality. During data collection, double checking the questionnaires was employed by the principal investigator.

A one-day training was conducted for all research team members on data collection methods, tool pretesting and reflection. The contents of the training included privacy assurance, confidentiality, interview techniques, and reviews of the study protocol and questionnaire. Research team members were also equipped with quality control skills such as rechecking and reviewing the questionnaires after administration as well as resolving issues that might arise during the fieldwork.

### Data management and data analysis

The data were entered in Epi INFO version 7 and transferred in to statistical package for social science (SPSS) Version 20. The data were cleaned and analyzed using SPSS for windows version 20 (Illinmoise Chicago). Frequency distribution table was used to show the distribution of variables. Percentages and table were used describe result of the study. Chi square was used to examine the relationship between minimum dietary diversity and associated factors. *P* < 0.05 was considered statistical significance. Bivariate and multivariabe logistic regression analysis were employed. Variables that had* P* value less than 0.25 in the bivariate model were entered into the multivariable regression model. Different factors were regressed with minimum dietary diversity as dependent variable to isolate its independent predictors. Model fitness was checked using Hosmer Lemeshaw test at *P* > 0.05. Multicolliniary was checked using standard error ≥ 2.0. The results are presented using adjusted Odds Ratios and 95% confidence intervals.

Wealth index was generated by asking durable assets including radio, TV, mobile phone, hand cart, plough plow. Ownership of each asset was given a score of “1” and non ownership was given a score of “0”. Principal component analyses was conducted to generate wealth index after checking all assumptions such as sample adequacy (KMO ≥ 0.5, antiimage ≥ 0.5 and communality ≥ 0.5, Bartlets test of Sphericity (*P* < 0.5) and absence of variable with complex structure. The wealth index score was then rank ordered into tertile and the highest tertile was taken as rich while the two lowest tertile combined were taken as poor.

## Results

Out of the total 530 sampled mothers/care takers who had children age from 6-23 months, 512 of participated in the study with the response rate of 96.6%. The mean (+ sd) age of mothers/caregivers was 32(± 6.1) years. Out of the total respondents (mothers) interviewed, over half (55.7%) of them were between the age group of 25–34 years (Table 1).

**Table 1:** Socio-Demographic and Economic Characteristics Mothers Having Child Aged 6–23 Months in Enebsie Sar Midir Woreda, March 2020

Characteristics	Frequency	Percentage
**Age (*n*** ** = 512)**		
15–24	47	9.2
25–34	285	55.7
> 35	180	35.2
**Marital Status (*n*** ** = 512)**		
Married	504	98.4
Unmarried	8	1.6
**Residence**		
Urban	66	12.9
Rural	446	87.1
**Educational Status (*n*** ** = 512)**		
Illiterate	400	78.1
Elementary	69	13.5
Secondary	22	4.3
Higher education	21	4.1
**Current Occupation (*n*** ** = 512)**		
House worker/housewife	398	77.7
Office worker	27	5.3
Daily laborer	27	5.3
Merchant	10	2.0
Farmer	50	9.8
**Wife/Husband occupation (*n*** ** = 512)**		
House worker/housewife	52	10.2
Office worker	38	7.4
Daily laborer	10	2.0
Merchant	22	4.3
Farmer	390	76.2
**Household Family Size (*n*** ** = 512)**		
< 4	71	13.9
≥ 4	441	86.1
**Number of under-five children (*n*** ** = 512)**		
< 2	255	49.8
≥ 2	257	50.2
**Child Age (in month) (*n*** ** = 512)**		
6–11 months	152	29.7
12–17 months	193	37.7
18–23 months	167	32.6
**Availability of Media Source in Household (*n*** ** = 512)**		
Yes	153	29.9
No	359	70.1
**Exposure to available media Sources (*n*** ** = 512)**		
Yes	151	29.5
No	361	70.5
**Available farmland (*n*** ** = 512)**		
Yes	449	87.7
No	63	12.3
**Cultivating vegetables (*n*** ** = 512)**		
Yes	480	93.8
No	32	6.3
**Available livestock (*n*** ** = 512)**		
Yes	444	86.7
No	68	13.3
**Having cow milk and feed the child (*n*** ** = 512)**		
Yes	130	25.4
No	382	74.6
**Mother involved in decision-making (*n*** ** = 512)**		
Yes	508	99.2
No	4	.8
**Agro ecology (*n*** ** = 512)**		
Dega	116	22.7
Woyinadega	264	51.6
Kola	132	25.8
**Wealth index (*n*** ** = 512)**		
Poor	359	70.1
Rich	153	29.9
**Number of Animals (*n*** ** = 512)**		
0–5	184	35.9
6–10	117	22.9
> 10	211	41.2

### Food groups consumed in 24 h

**Fig. 1:**
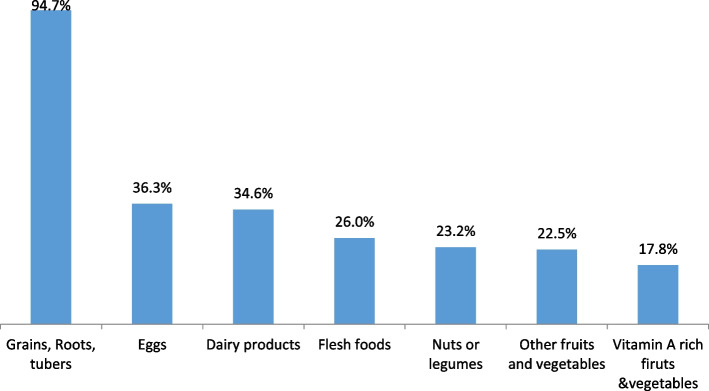
Types of food groups consumed by children aged 6–23 in Enebsie Sar Midir Woreda, march 2020 (*n* = 512)

The study showed grain, roots and tubers were the most commonly consumed food items 24 h prior to the survey followed by eggs; i.e., it is consumed by 485 (94.7%) and 187 (336.5%), respectively. However, the consumption of vitamin A rich fruits and vegetables was low. From the total children only 91 (17.8%) consumed Vitamin A rich fruits and vegetables (Fig. 1).

### Minimum dietary diversity

All mothers included in this study had started giving solid, semi-solid or liquid foods for their children and all of the children had received complementary foods before the day of the study. The frequency of children who had received minimum dietary diversity within 24 h previous data collection was 18.2%.( CI; 14.8, 21.7). Two hundred eighteen (42.6%) of the sampled children took one types of food at the time of survey on the 24-h recall followed by two meals (26.4%) while the frequency of children who consumed three meals was 12.9%.

From all variables recruited, twenty-two variables were significantly associated with minimum dietary diversity on bivariate logistic regression analysis. Bivariate analyses showed that significantly higher proportion (88.1%) households did not practice minimum dietary feeding than household residue in urban area (12.9%), *P* < 0.001.

Educational status of the mother was significantly associated with minimum dietary practice of mothers who completed higher education were likely to feed diversified diet than illiterate mothers (78.2%), (*P* < 0.001). Similarly, 5.3% of mothers who were office workers practiced minimum dietary diversity compared to house wife or house workers. A larger proportion (86.1%) of children from households greater than four family size also reported that they did not get diversified food compared to households with less than four family size (13.9%), (*P* < 0.001).

Minimum dietary feeding practice was significantly higher among households with availability of Media Source at home (29.9), compared to those children’s from household with no availability of media source at home (70.1%), (*P* < 0.001). A significantly (*P* < 0.001) higher proportion (50.2%) of children form households with number of under-five children in the household of greater than two did not get proper diversified diet, compared to those form households with number of under-five children’s less than two (49.8%).

From a total of twenty-two variables considered for the bivariate analysis, only cultivating vegetables, having cow milk, availability of farm land and number of animals were significantly associated with minimum dietary diversity in the multivariable logistic regression model.

On multivariable logistic regression model, after adjusting for other variables, those mothers who from households that cultivate vegetables were 3.2 times (AOR = 3.2, 95%CI: 1.05, 9.8) more likely to feed their children with optimum dietary diversity compared to those who do not cultivate vegetables. Those who had cow’s milk in the household were 17.27 times (AOR = 17.27, 95%CI: 6.73, 44.44) more likely to feed their children with optimum dietary diversity. Women (parents) who had farmland were 10.1(AOR = 10.15, 95%CI: 1.78, 57.93) times more likely to feed their children. Those women (parents) who had number of animals six to ten were 6.46 times more likely to feed their children with optimum dietary diversity compared to those who had number of animals less than five( AOR = 6.46, 95%CI: 1.97, 21.12 ). The likelihood of feeding optimum dietary diversity to their children was 15 times more (AOR = 15.12, 95%CI:4.31, 53.05 ) among mothers (parents) who had number of animals greater than ten compared to those who had number of animals less than five (Table 2).

**Table 2:** Bivariate and multivariate logistic regression analyses predicting minimum dietary diversity among children aged 6–23 months in Enebsie Sar Midir Woreda, 2020 (*n* = 512)

Characteristics	Minimum DDS		COR (95%CI)	AOR (95%CI)
	**Yes**	**NO**		
**Marital Status (*n*** ** = 512)**				
Married	89	425	1.00	1.00
Unmarried	4	4	0.21 (0.05,0.87)*	0.2 (0.01, 4.06)
**Residence (*n*** ** = 512)**				
Urban	50	16	1.00	^1.00^
Rural	43	403	29.29(15.37,55.81)***	0.98(0.05,21.05)
**Educational Status (*n*** ** = 512)**				
Higher education	93	2	1.00	^1.00^
Illiterate	36	364	96.0(21.5,43)***	0.24(0.005,11.03)
Primary	21	48	21.7(4.63,101)***	0.21(0.005,8.84)
Secondary	17	5	2.79(0.47,16.3)	0.04(0.001,2.21)
**Current occupation (*n*** ** = 512)**				
House worker/housewife	55	343	1.00	1.00
Office worker	24	3	0.02(0.06,0.069)***	0.29(0.01,6.56)
Daily laborer	5	22	0.70(0.25,1.94)	0.82(0.16,4.12)
Merchant	6	4	0.10(0.29,0.39)***	1.19(0.15,9.48)
Farmer	3	47	2.51(0.75,8.35)	0.59(0.09,4.05)
**Your wife/husband Current occupation (*n*** ** = 512)**				
House worker/housewife	8	44	1.00	1.00
Office worker	36	2	0.010(0.002,0.05)***	0.07(0.004,1.23)
Daily laborer	0	10	293,722,702(0.000,)	134,750,446.6(0.000)
Merchant	12	10	0.15(0.05,0.47)***	1.74(0.17,18.09)
Farmer	37	357	1.73(0.76,3.96)	0.61(0.11,3.49)
**Household family size (*n*** ** = 512)**				
< 4	20	51	1.00	^**1.00**^
> = 4	73	368	1.97(1.11,3.51)***	0.69(0.22,2.23)
**Number of under 5 children’s (*n*** ** = 512**)				
< 2	80	175	1.00	^**1.00**^
> = 2	13	244	8.58(4.63,15.91)***	2.12(0.82,5.46)
**Availability of media source at household (*n*** ** = 512)**				
Yes	63	90	1.00	1.00
No	30	329	7.67(4.68,12.57)***	0.001(0.001)
**Exposure to available media sources (*n*** ** = 512**)				
Yes	63	88	1.00	1.00
No	30	331	7.89(4.81,12.9)***	313(0.000)
**Available Farmland (*n*** ** = 512)**				
No	52	11	1.00	1.00
Yes	41	408	47(22.77,97.1)***	10.15(1.78,57.95)***
**Cultivating vegetables (*n*** ** = 512)**				
No	80	400	1.00	1.00
Yes	13	19	3.42(1.62,7.21)***	3.2(1.05,9.8)*
**Available livestock (*n*** ** = 512)**				
Yes	43	401	1.00	^**1.00**^
No	50	18	0.04(0.02,0.07)***	1.07(0.05,23.44)
**Having cow milk and feed the child (*n*** ** = 512)**				
No	57	325	1.00	1.00
Yes	36	94	2.18(1.36,3.52)***	17.27(6.73,44.44)***
**Agroecology (*n*** ** = 512)**				
Dega	9	107	1.00	^**1.00**^
Woyinadega	74	190	0.22(0.1,0.45)***	1.15(0.4,3.3)
Kola	10	122	1.03(0.4,2.62)	0.68(0.22,2.16)
**Wealth Index**				
Poor	28	331	1.00	1.00
Rich	65	88	0.12(0.69,0.19)***	1.06(0.43,2.65)
**Number of Animals (*n*** ** = 512)**				
0–5	76	108	1.00	1.00
6–10	7	110	11.06(4.88,25.07)	6.46(1.97,21.12)**
> 10	10	201	14.14(7.03, 28.47)	15.12(4.31,53.05)***

*p-value < 0.05, **p-value < 0.01, ***p-value < 0.001

## Discussion

The results showed that 18.2 (95% CI; 14.8, 21.7) of the children aged 6–23 months had four or more food groups meeting the minimum requirement of dietary diversity. This result is similar to that of research conducted in southern Ethiopia [24], In this study, the consumption of vitamin-rich fruits, dairy products, meat, vegetables, and other fruits was low. This was similar with the studies done in Ethiopia [25]. The possible reason might be cereals, grains and tubers are the commonest products in the study area, And the family or caregivers put the food stuffs (vitamin rich fruits, dairy products, flesh, and vegetables and other fruits) on the market for sale, they won’t let the child eat them. in which commonly consume those food items they cultivated and accessed from the market in low cost.

Although the result of the current study is greater than that of the EDHS 2016 national survey and some other national studies [19]. Only 13% of youngsters in Sinan Woreda exceeded the minimum dietary diversity requirement, according to research [21]. The finding is lower than other studies done in Addis Ababa, Ghana, Indonesia, and Myanmar [11, 16, 26, 27].

The difference in food diversity between the current study and other researches could be attributable to paternal educational level, socioeconomic inequalities, and geographical variation. The low practice of giving different types of food after six months, income shortage to buy foods that are not available at home, and family habit or culture, i.e. there are no specific types of food prepared for children and also those foods that are not available at home, are all possible reasons for low dietary diversity practices in the study area. Animal source foods are not also commonly used as routine types of food instead they get consumed during holidays or events like wedding.

This study found out that cultivating vegetables, availability of cow milk, available farmland and number of animals were factors associated with meeting MDD.

The likelihood of feeding children with optimum dietary diversity was more among mothers who cultivated vegetables at household. This might be due to easy accessibility of vegetables. Mothers or care givers who had available cow’s milk at household level were more likely to feed diversified food group than those who had not. The possible reason might be the household that have cow’s milk, feed their child milk and milk products in addition to the common diet. Similar result has been reported by the study conducted in Ethiopia [21].

Unlike previous studies which was conducted in Ethiopia and Ghana [7, 16] there is no association between maternal education and minimum dietary diversity in this study. There is no association between wealth index, maternal age and minimum dietary diversity in this study. This result is supported by similar study conducted in Ethiopia [28], and also this study finding didn’t show any association between maternal age and minimum dietary diversity unlike the study conducted in Robe Town, Bale Zone [25]. The results have ramifications for the research area’s efforts to improve child feeding. Improved land and animal ownership, consumption of cow’s milk in the home, and intake of readily available vegetables were all strongly linked to MDDS, which is consistent with the national food and nutrition policy as well as the nutrition-sensitive agricultural plan.

This study acknowledges the common limitations of cross sectional study. Effect of seasons on the minimum dietary diversity could not be assessed due to cross-sectional nature of the study. Accurately reporting participants; past feeding dietary habit could be a problem due to memory laps (recall bias) which was tried to be minimized by probing them during data collection. Including qualitative data would have added to the findings by capturing the how’s and whys behind the numbers related to cultural issues in this study area, it was not done in this study.

## Conclusion

The study showed important gap to meet optimal minimum dietary diversity. Based on findings of this study, the overall prevalence of optimum dietary diversity score among children aged 6– 23 months in Enebsie Sar Midir Woreda is low compared with other countries. To achieve the optimal minimum dietary diversity feeding practice for all children aged between 6 and 23 months in the study area it needs more work. Availability of cow’s milk in the household, cultivating vegetables, availability of farm land and number of animals was significantly associated with optimal dietary diversity feeding practice of children aged 6–23 months. In this study consumption of fruits and vegetables was relatively low. The Federal Ministry of Health should provide nutrition guidance on IYCF for mothers or caregivers, as well as practice infant and young child feeding according to community guidelines and promote IYCF. Farmers should be educated on how to use their land to cultivate a variety of commodities, such as fruits and vegetables.

## Data Availability

Data used for this study is available upon a reasonable request to the corresponding author.
